# Necrotizing Skin and Soft Tissue Infection Due to *Syncephalastrum* Species and *Fusarium solani* Species Complex Following Open Tibia Fracture

**DOI:** 10.3390/diagnostics12051163

**Published:** 2022-05-07

**Authors:** Vasiliki Mamali, Christos Koutserimpas, Kassiani Manoloudaki, Olympia Zarkotou, George Samonis, Georgia Vrioni

**Affiliations:** 1 Department of Clinical Microbiology, “Tzaneio” General Hospital of Piraeus, 18536 Piraeus, Greece; mamalivasiliki@outlook.com.gr (V.M.); olyzar@hotmail.com (O.Z.); 2 Department of Orthopaedics and Traumatology, “251” Hellenic Air Force General Hospital of Athens, 11525 Athens, Greece; chrisku91@hotmail.com; 3 Department of Pathology, “Tzaneio” General Hospital of Piraeus, 18536 Piraeus, Greece; kassy.manol@yahoo.com; 4 Department of Medicine, University of Crete, 71500 Heraklion, Greece; 5 Department of Microbiology, Medical School, National and Kapodistrian University of Athens, 11527 Athens, Greece; gvrioni@med.uoa.gr

**Keywords:** necrotizing infection, mucormycosis, fusariosis, fungal infection, mucorales, *Syncephalastrum* spp., *Fusarium solani*, skin and soft tissue infection

## Abstract

Fungal necrotizing skin and soft tissue infection (NSSTI) represents a rare clinical entity. An extremely rare case of NSSTI, following an open tibia fracture in a 36-year-old male caused by both *Syncephalastrum* spp. and *Fusarium solani* species complex (SC) is presented. The infection was diagnosed through direct microscopy, cultures and histology. The disease had a long course. The patient underwent a total of seven consecutive surgical debridements, while proper and timely antifungal treatment was initiated and included liposomal amphotericin B and voriconazole. He gradually recovered and 4 years later he is completely functioning and healthy. Invasive fungal infections are well-documented causes of high morbidity and mortality in immunocompromised individuals, whereas in immunocompetent hosts, trauma-related fungal infections have also been reported. It is of note that *Syncephalastrum* spp. has very rarely been identified to cause infection in immunocompromised or immunocompetent hosts, whereas *Fusarium* spp. has rarely been involved in skin necrotic lesions in non-immunocompromised individuals. A high suspicion index, especially in necrotic lesions in trauma patients, is pivotal for early diagnosis, which may lead to lower mortality as well as lower amputation rates. Definite diagnosis through microscopy, histology and/or cultures are of paramount importance, whereas PCR testing may also be extremely useful.

## 1. Introduction

Skin and soft tissue infections are among the most common bacterial infections, accounting for approximately 10% of hospital admissions due to infections in the USA [1,2]. Skin and soft tissue infections represent clinical entities of variable presentations, etiology and severity that involve microbial invasion of the layers of the skin and underlying soft tissues, ranging from mild to serious life-threatening infections. They are classified as simple (uncomplicated) or complicated (necrotizing or non-necrotizing) and can involve the skin, subcutaneous fat, fascial layers and musculotendinous structures [1,2,3].

Necrotizing skin and soft tissue infection (NSSTIs) refer to different syndromes of gangrenous infection of the skin and subcutaneous tissues. They are rapidly progressing through the skin and the soft tissues leading to tissue necrosis. The disease may extend beyond the fascia and include muscles as well. The main pathogens leading to this necrotizing entity are bacteria, whereas such fungal infections are rare [1]. The incidence of NSSTIs due to invasive group A *streptococcal* infections, which represent the most common causative organism in the United States, is estimated to be about 0.4 per 100,000 people [1,2]. However, the estimated incidence of NSSTIs in the general population remains unclear, due to the wide variability of the reported cases and series. The mortality of this severe infection remains high, at approximately 25–30%, in spite of advances in therapeutic management, whereas in recent years, mortality has only exhibited a decrease to just over 20% [2]. Case fatality rates remain extremely high in cases where the infection is accompanied with sepsis and systematic shock, and/or host factors such as advanced age, comorbidities or immunocompromised state [1,2].

The anatomical sites of these infections may also define the terminology. The term “Fournier’s gangrene” is used to describe necrotizing fasciitis of the perineum, which is generally polymicrobial [2,4]. Diabetic foot infections that compromise microvasculature and may evolve to necrotizing pattern are usually polymicrobial with anaerobic milieu [5]. Terminology and classification may also be based upon the necrosis depth. Necrotizing cellulitis involves the dermis and subcutaneous tissue. Necrotizing fasciitis involves the fascia, whereas pyomyositis or myonecrosis describes involvement of the muscle fascicle without necessarily overlying skin infections [4].

Filamentous fungi such as *Mucorales*, *Aspergillus* spp., *Fusarium* spp. and *Scedosporium* spp. are well-recognized in causing invasive fungal infections in immunocompromised patients [6,7,8]. However, in immunocompetent hosts, cutaneous mucormycosis has been also reported [8]. Penetrating trauma with direct inoculation of fungal spores is the most common mode of transmission, resulting in localized necrosis or deep-seated infection, and is rarely disseminated in patients with no severe underlying disease [8].

Regarding mucormycosis, *Rhizopus* is the most common genus followed by the genera *Mucor* and *Lichtheimia*. *Syncephalastrum* is considered the least pathogenic mucoralean mold, rarely related with disseminated infection [8,9]. Fusarial skin involvement with localized necrotic lesions has rarely been reported in non-immunocompromised patients [10].

An extremely rare case of NSSTI following an open tibia fracture in a 36-year-old male caused by *Syncephalastrum* spp. and *Fusarium solani* species complex (SC) is presented, aiming to report infection due to a rather rare species and even more rare species combination causing life-threatening disease.

## 2. Case Presentation

A 36-year-old male, with an unremarkable medical history, was brought to the Emergency Department due to a motorcycle accident. Upon presentation he was hemodynamically stable (blood pressure: 120/70 mmHg, heart rate: 90 beats per min).

The patient was initially treated according to the Advanced Trauma Life Support guidelines (ABCs). The patient was suffering a Gustilo-Anderson type IIIA open tibia fracture. He was urgently taken into the operating room where surgical debridement was performed and a hybrid external fixation was placed. Although there was extensive soft-tissue damage, adequate tissue for flap coverage existed and the wound was closed.

**Figure 1:**
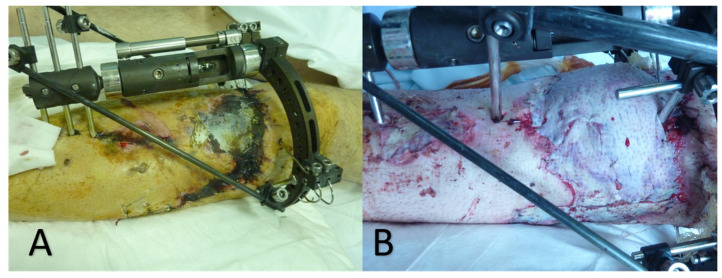
Skin necrotic lesion, located at the anterior tibia, arising from the surgical wound (**A**) ten days after external fixation of the open complicated tibia fracture and (**B**) thirty days after the initial surgical debridement.

The patient was commenced on intravenous cefuroxime 750 mg × 3 daily, gentamycin 500 mg × 2 daily and clindamycin 600 mg × 1 daily. He was hemodynamically stable and afebrile. On the 10th postoperative day, a skin and soft tissue necrotic lesion located at the anterior tibia was developed, arising from the surgical wound (Figure 1).

**Figure 2:**
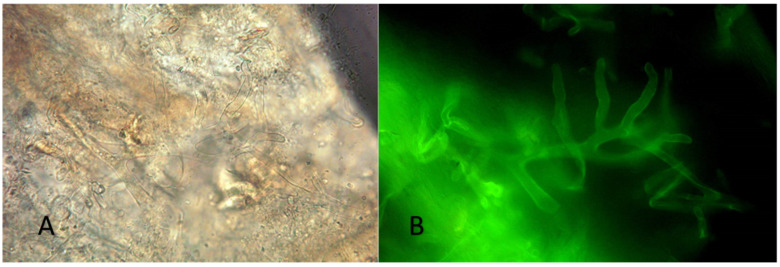
Direct microscopy: wet tissue mount examined with KOH and (**A**) Blankophor-P (**B**) broad, non- septate, ribbon- like hyphae with right-angle branching, characteristic of *Mucorales*.

**Figure 3:**
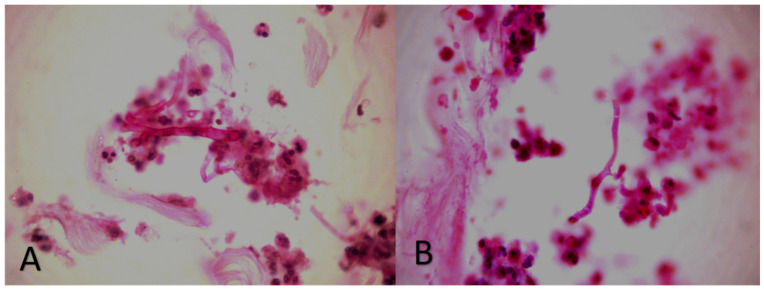
Histopathological examination: intense inflammatory infiltration consisting of neutrophils with many (**A**) hyalines, branched, aseptate hyphae presumptive *Mucorales* and (**B**) hyalines, branched, septate hyphae, presumptive hyphomycetes other than *Mucorales* (stain: haematoxylin- eosin, magnification: ×40).

**Figure 4:**
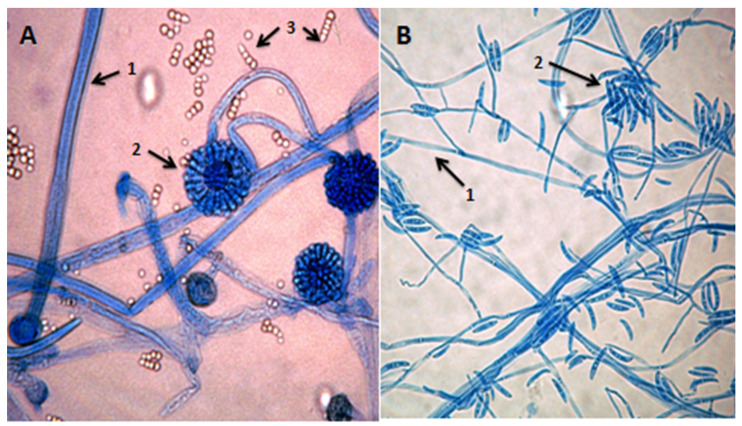
Microscopic examination of cultured fungi at 40× magnification (some of the fungus colony was picked up with adhesive tape, placed on clean glass slide and covered with a slip). (**A**): Microscopic image of *Syncephalastrum* spp. stained with lactophenol cotton blue: (1) broad, non-septate hyphaes, (2) sympodially branching sporangiophores with terminal vesicles bearing finger-like merosporangia directly over their entire surface, and (3) globose to ovoid, smooth-walled sporangiospores (merospores). (**B**): Microscopic image of *Fusarium solani* SC stained with lactophenol cotton blue: (1) hyaline hyphaes and (2) septate macroconidia slightly curved, hyaline and broad.

At that point in time, extensive surgical debridement was performed and multiple tissue specimens from skin and subcutaneous tissues were microscopically and histologically examined as well as cultured. Wet tissue mount examined with KOH and Blankophor-P, demonstrated broad, non-septate, ribbon-like hyphae with right-angle branching, characteristic of *Mucorales*. Additionally, thin, septate hyphae, consistent with hyaline molds other than *Mucorales*, were also observed (Figure 2). Tissue cultures on Sabouraud dextrose agar yielded *Syncephalastrum* spp. and *F. solani* SC. Matrix-assisted laser desorption ionization time of flight mass spectrometry (MALDI-TOF) on a Microflex LT (BrukerDaltonics, Bremen, Germany) platform confirmed the identification of the isolates as *S. rasemosum* and *F. solani*. The given log score for both fungal pathogens were >2.0 (2.3 for *S. rasemosum* and 2.2 for *F. solani*. Susceptibility testing of *Syncephalastrum* spp. showed low MICs values for amphotericin B (MIC = 0.25 mg/L), whereas susceptibility testing for *F. solani* SC yielded high MICs values for both amphotericin B and voriconazole (MICs equal to 4 mg/L) (SensititreYeastOne; Thermo Fisher Scientific, Cleveland, OH, USA). Histopathological examination showed an intense inflammatory infiltration mainly consisting of neutrophils and many hyalines, branched, aseptate and septate hyphae, stained with both haematoxylin-eosin (H&E) and periodic acid-Schiff (PAS) (Figure 3). Microscopic examination, as well as skin and soft tissue cultures, did not identify any other organisms, such as bacteria or cocci (Figure 4). Blood cultures were negative.

Immediately after the suggesting fungal infection direct microscopy, antifungal treatment was initiated. Liposomal amphotericin B (5 mg/kg/day) combined with voriconazole (200 mg twice daily) were administered intravenously. The treatment did not cause serious side effects, apart from vision changes and sensitivity to light, attributed to the voriconazole. In total, seven successive surgical debridements were required because both molds were repeatedly recovered from follow-up tissue cultures. Finally, a vacuum-assisted, negative pressure device was used to promote revascularization of the area.

On the 30th postoperative day (from the initial surgical debridement), cultures became negative; the patient showed marked clinical improvement and the trauma was evaluated, and it was decided the patient would receive an autologous skin graft (Figure 1). The antifungal treatment has been continued until a week after all signs and symptoms of the infection had disappeared (the total duration of antifungal treatment was 40 days). Four years later, the patient is fine—fully functioning and without symptoms or signs of the infection.

## 3. Discussion

NSSTI represents a rapidly progressing skin infection characterized by tissue necrosis and accompanied by severe systemic toxicity [4]. This infection is known from the Hippocrates era, and it is accompanied with increased mortality and amputation rates [11].

The rapidly progressive necrosis involving this infection is due to the cycle of fulminant infection consisting of toxin production, cytokine activation, microthrombosis and ischemia, tissue dysfunction and death, and in turn, further dissemination of the infection [1,12]. More specifically, regarding tissue necrosis due to Mucorales infection, the disease occurs rapidly and once the filamentous growth has been initiated, it leads to angioinvasion (a hallmark of infection enabling the hematogenous dissemination of the disease), thrombosis and tissue necrosis [9]. Surgical removal of necrotic tissues is the cornerstone of therapeutic management, since antimicrobial treatment penetrates dead and dying tissue poorly [1,12,13]. Initial empirical treatment should include broad-spectrum agents covering polymicrobial infections. This should include a methicillin-resistant *Staphylococcus aureus* (MRSA), an active agent such as vancomycin, daptomycin, linezolid or ceftaroline, as well as a broad-spectrum agent that works against gram-negative pathogens such as piperacillin-tazobactam, ampicillin-sulbactam, ticarcillin-clavulanate, extended-spectrum cephalosporins or carbapenems. Anaerobes should also be covered with an agent such as metronidazole or clindamycin. Empirical antifungal therapy is not recommended, but an appropriate antifungal agent should be added upon visual evidence on stains or growth in blood or operative cultures of fungal elements such as *Candida* or *Mucorales* spp. [12,13].

In the majority of NSSTI cases, a causative pathogen is isolated from the infected tissue, whereas about 35% of blood cultures are also positive [7]. This infection in most cases is polymicrobial, whereas the most frequent isolated organisms are: *Staphylococcus* spp., *Streptococcus* spp., *Bacteroides* spp., *Enterobacterales* (*Escherichia coli* and *Klebsiella* spp.), and *Pseudomonas* spp. *Vibrio vulnificus*, *Aeromonas hydrophila* and *Clostridium perfringens* are also relatively common [1,9,11]. Fungal NSSTI is extremely rare, with only a few reports so far in the literature, mostly in immunocompromised patients [11].

The present report presented an extremely rare case of NSSTI following an open tibia fracture in a 36-year-old male caused by both *Syncephalastrum* spp. and *F. solani* SC. The patient had an unremarkable medical history and the spread of both molds should be considered to be through direct inoculation, most probably at the time of the motorcycle accident.

Mucormycosis (Zygomycosis) represents a spectrum of emerging infections caused by ubiquitous, opportunistic fungal pathogens of the order *Mucorales*, such as *Mucor* spp., *Rhizopus* spp., *Rhizomucor* spp., *Lichtheimia* spp. and *Syncephalastrum* spp., and that may be proven severe [8,9]. The main predisposing factors of mucormycosis include hematological malignancies, organ transplantation, human immunodeficiency virus/acquired immunodeficiency syndrome (HIV/AIDS), hematopoietic stem-cell transplant, lymphoid malignancies, neutropenia, hereditary immune deficiencies, immunosuppressive medications, diabetes mellitus and intravenous drug abuse [8,14]. *Syncephalastrum* species are found in the environment and tropical soil and rarely cause human infection. When clinically significant, they are usually implicated in cutaneous infections and onychomycos. Only a few reported cases involve sites other than skin and nail tissues [9,14]. *Syncephalastrum* species rarely result in pneumonia. Similar to other species of Mucormycosis, *Syncephalastrum* may also lead to rhino-cerebral or rhino-orbital-cerebral infection, while one case of intra-abdominal infection has been reported [14].

The infection is usually acquired by inhalation of sporangiospores, occasionally by ingestion of contaminated food or traumatic inoculation [15]. It is of note that cutaneous mucormycosis is the third most common site of infection (19%), followed by the sinuses (39%) and the lungs (24%) [16]. It is of note that in some geographical areas, such as India, the skin localization of the infection may be the second most common one [17]. However, 44% of the cutaneous infections are complicated by deep-seated or disseminated infections [5,16,18,19], especially in immunocompromised patients, when progression from a painless papule to necrotizing infection may occur either surreptitiously or as fulminant process. Necrosis has been documented in up to 60% of these cases [20].

Despite the fact that other filamentous fungal infections target immunocompromised hosts, the majority of patients reported with cutaneous mucormycosis had no other underlying conditions [16]. Disruption of the skin barrier, due to burns, trauma or surgery, is the main risk factor for developing cutaneous mucormycosis [21]. Earthquakes and other natural disasters (tornados, tsunamis, volcanic eruptions) have also been linked with cases of cutaneous mucormycosis, because of penetrating wounds or crush injuries, that have become contaminated with moist organic matter [8,9]. Moreover, invasive mold infections have been reported following combat-related traumatic injuries among military personnel [22]. This could be the mechanism of the infection in the reported case as well, since traffic accidents are usually associated with widespread tissue damage, which is easily contaminated with soil [8,9,23].

*Syncephalastrum*, belonging to the order *Mucorales*, is a ubiquitous saprophytic fungus, commonly found in soil, especially in climates with high humidity [24]. It is usually presented as a colonizer and rarely as an etiological agent of human infections in immunocompromised or immunocompetent hosts [15,25,26,27,28]. Its colonies grow very rapidly and fill a 90 mm plate completely in 48 h. The color of colonies, from the front, is initially white and turns to dark gray then black in time due to sporulation, whereas from the reverse, it is pale or yellowish-brown. Microscopically, *Syncephalastrum* spp. Has a ribbon-like aseptate, branched fungal hyphae and sporangiophores, which terminate in swollen vesicles with radial merosporangiae filled with a linear series (chains) of sporangiospores. *Syncephalastrum* differs from *Aspergillus* by the presence of merosporangia and absence of phialides [25,26,27,28,29].

*F. solani* is a species complex of at least 50 closely related filamentous fungi in the division *Ascomycota*, family *Nectriaceae* [10]. It is the anamorph of *Nectriahaematococca* [30]. It is a common soil fungus and colonist of plant materials. Colonies of *F. solani* SC are woolly to cottony with cream to white aerial mycelium and a cream reverse. Microscopic examination of colonies shows septate, hyaline hyphae, macroconidia moderately curved, thick-walled, usually three to five septate and microconidia one to three-celled, occurring in clusters at the tip of conidiophores [10,30].

The most common clinical manifestations of fusariosis in immunocompetent hosts are onychomycosis, keratitis and allergy [31]. In these type of patients, cutaneous infections are characterized by preceding skin breakdown, localized involvement, slow pace of progression and good response to therapy [32]. *F. solani* SC is implicated in plant, as well as in human diseases, and is responsible for nearly 60% of fusariosis cases, isolated notably from infections of the cornea of the eye [10,33]. *Fusarium* spp. has rarely been involved in skin necrotic lesions in non-immunocompromised individuals [30]. In addition, mixed infections of *F. solani*-*Mucorales* are extremely rare, and according to the existing literature, have been described once in a young patient treated for acute myelogenous leukemia [34].

Clinical suspicion should be raised in rapidly necrotizing traumas and should be confirmed with a tissue-based laboratory investigation. Direct examination of wet tissue mount with KOH, preferably with the addition of a fluorescent whitening agent, such as Blankophor P, is important for prompt diagnosis. Regarding identification of *Mucorales* by MALDI-ToF MS, this is a reliable and rapid method for the identification of most of the human pathogenic *Mucorales* to the species level. Moreover, as mentioned by Schwarz P. et al., for some species, similar to *Syncephalastrum*, for which only a low number of isolates is included in the database, it is of major importance to include several isolates of each species into the database to maximize the possibility of reliable identification [35]. Furthermore, histopathology and culture-based techniques are affirmed to be cornerstone of prime diagnosis of fungal infections. Surgical debridement along with proper antimicrobial and/or antifungal treatment represents the proper management of NSSTI [3].

Early recognition and urgent initiation of treatment of NSSTI are of utmost importance for minimizing morbidity and mortality rates [1]. The clinical signs that should raise the suspicion of such a severe infection include swelling and erythema, whereas the most consistent clinical finding is disproportionate pain. However, it can often be difficult to discern a necrotizing process from a simple cellulitis [2,4].

Delayed (even for a few hours) surgical intervention has been associated with increased mortality, and high suspicion of this severe infection should lead promptly to patients in the operating theater for removal of all necrotic tissues [36]. Involved tissues should be resected meticulously until there is no macroscopical evidence of infection. Multiple surgical debridements may be required [37]. The reported patient underwent a total of seven successive such surgical interventions. The “second look” is usually performed 12–24 h after the initial surgery. Typically, an average of five to forty such “surgical sessions” may be necessary [25]. In cases of wide, aggressive “disfiguring” debridements, multidisciplinary surgical approaches may be necessary, including urology for perineal wounds or wounds involving the penis or scrotum, and plastic surgery for complex reconstruction or muscle flap reconstruction and/or orthopedics for osseous involvement [3].

Following aggressive surgical debridement and successful antimicrobial treatment, reconstruction with rotational flaps or skin graft techniques may be necessary, as this was performed in the presented patient [36]. The widespread use of vacuum-assisted closure devices in everyday clinical practice provides consistent and easy nursing care, the suctioning of soft tissue edema and the promoting of granulation tissue [3]. Furthermore, the newer vacuum-assisted closure devices offer continuous wound irrigation, which may be beneficial in wounds after surgical debridement. Negative pressure dressings can provide dermotraction to limit the wound size and facilitate closure. It is of note that rehabilitation and physical therapy may be necessary in cases of repeated reconstructive surgeries; however, these operations may change the patient’s quality of life dramatically [3,36].

## 4. Conclusions

Definite and prompt diagnosis of NSSTI is of utmost importance for minimizing the disease’s mortality and amputation rates. A high suspicion index, especially in necrotic lesions in trauma patients, is pivotal for early diagnosis and must be confirmed with a tissue-based laboratory investigation. Definite diagnosis through microscopy, histology and/or cultures are of paramount importance, whereas PCR testing may also be extremely useful, especially in doubtful cases and those where antifungal treatment is required. Management involves proper antimicrobial and/or antifungal treatment and successive surgical debridements to the point of healthy tissue and negative cultures.

## Data Availability

Not applicable.
